# Transcriptional Heterogeneity and the Microbiome of Cutaneous T-Cell Lymphoma

**DOI:** 10.3390/cells11030328

**Published:** 2022-01-19

**Authors:** Philipp Licht, Volker Mailänder

**Affiliations:** 1 Dermatology Clinic, University Medical Center of the Johannes Gutenberg-University Mainz, Langenbeckstraße 1, 55131 Mainz, Germany; plicht@uni-mainz.de; 2 Max Planck Institute for Polymer Research, Ackermannweg 10, 55128 Mainz, Germany

**Keywords:** cutaneous T-cell lymphoma (CTCL), mycosis fungoides (MF), Sézary syndrome (SS), transcriptome, microbiome, heterogeneity

## Abstract

Cutaneous T-Cell Lymphomas (CTCL) presents with substantial clinical variability and transcriptional heterogeneity. In the recent years, several studies paved the way to elucidate aetiology and pathogenesis of CTCL using sequencing methods. Several T-cell subtypes were suggested as the source of disease thereby explaining clinical and transcriptional heterogeneity of CTCL entities. Several differentially expressed pathways could explain disease progression. However, exogenous triggers in the skin microenvironment also seem to affect CTCL status. Especially *Staphylococcus aureus* was shown to contribute to disease progression. Only little is known about the complex microbiome patterns involved in CTCL and how microbial shifts might impact this malignancy. Nevertheless, first hints indicate that the microbiome might at least in part explain transcriptional heterogeneity and that microbial approaches could serve in diagnosis and prognosis. Shaping the microbiome could be a treatment option to maintain stable disease. Here, we review current knowledge of transcriptional heterogeneity of and microbial influences on CTCL. We discuss potential benefits of microbial applications and microbial directed therapies to aid patients with CTCL burden.

## 1. Introduction

Cutaneous T-cell lymphomas (CTCL) are a group of skin homing neoplastic malignancies comprising approximately 10% of total non-Hodgkin lymphomas (NHL) with Mycosis fungoides (MF) as the most common entity [1]. Primarily presenting as an indolent disease course initially, there might be a sudden progression to advanced stages including extracutaneous site involvement and a 5-year overall survival drop from >80% to 44% in higher stages [2,3,4]. One third of patients progress to advanced stage MF within 10 years [5]. Because of the indolent character of early stage CTCL, typically only late-stage patients are directed to systemic therapies that can cause severe side effects [6]. Preselection of patients with a poor prognosis would allow these subjects to receive more intensive treatments possibly improving disease control and survival. Several clinical variables and biological markers are discussed as prognostic factors. However, these parameters are partially subjective and imprecisely specified or show conflicting results across studies [5]. Furthermore, diagnosis is challenging and requires a complex combination of factors. Early stages mimicking other inflammatory skin diseases, as well as a variety of clinical manifestations that can highly differ from the typical appearance, are aggravating diagnostic hurdles [5,7]. Hence, interobserver variation for MF diagnosis is considerable, resulting in a median delay of 3 years before the definitive diagnosis is made since the first patient presentation [5]. These challenges underline the need for better diagnostic and prognostic tools [8]. The variable nature of CTCL comes in hand with strong transcriptome heterogeneity (inter-patient and intra-tumoural) on both the bulk [9] and the single-cell level [10]. Genes with potentially prognostic value were identified [9,11], but results emphasize the need for personalized precision medicine [10,12]. Moreover, skin barrier dysfunction and upregulated virus entry pathways [12] may reflect increased susceptibility for skin infections already clinically observed in CTCL patients [13]. Many different pathogens were observed in infected CTCL patients and incidence rates vary strongly [14], following the diverse character of CTCL in that manner. Infectious agents are discussed to play a role in CTCL aetiology and pathogenesis [15]. Indeed, exogenous factors like bacterial toxins can enhance disease progression via unspecific activation and expansion of T-cells [16]. Especially the mechanisms of how *Staphylococcus aureus* can aggravate the progression of CTCL have been investigated [17]. In general, several microbiota or a dysfunctional microbial community can influence cancers and other skin diseases [18,19]. Regarding CTCL, the complex microbiome patterns possibly influencing the course of this disease have only recently begun to be investigated [20,21]. Genomic methods to study microbiota can provide deep insights into shifted microbial communities relating disease [19].

In this review, we discuss current knowledge of transcriptional heterogeneity and the microbiome of CTCL. We will first show that CTCL seems to arise from malignant cells in the blood, leading to intra-patient heterogeneity. We also indicate first evidence of microbial implications on disease progression in CTCL and how differential colonization could lead to inter-patient heterogeneity. Then, we outline transcriptional heterogeneity on the inter- and intra-patient level and underline aspects that could point to microbial influence. Next, we summarize current knowledge of the microbiome in CTCL. Finally, we discuss the interplay between the microbiome and transcriptome of CTCL and suggest possible microbial applications in CTCL.

## 2. T-Cell Receptor Clonality

**Figure 1:**
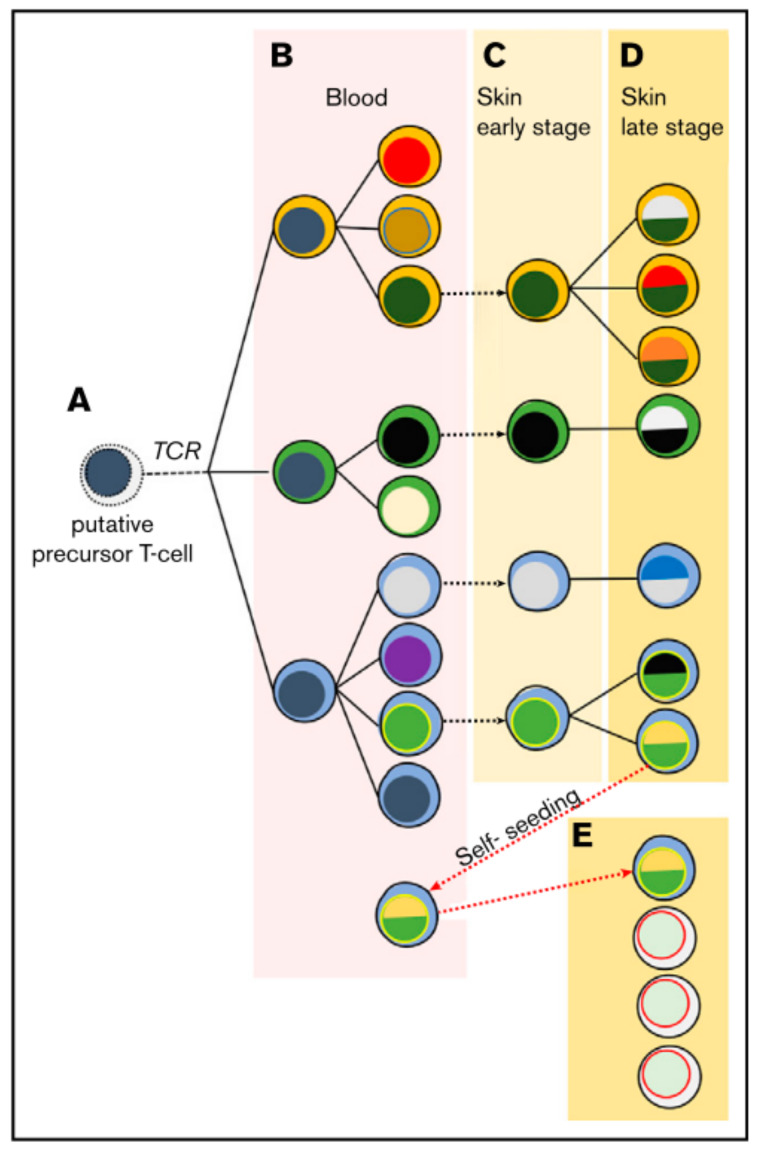
Suggested model of migrating malignant cells in CTCL. As a result of circulating and self-seeding malignant cells to other lesions, intra-patient heterogeneity may occur. (**A**) A putative precursor of a malignant T-cell undergoes malignant transformation before TCR-beta rearrangement, showing clonotypic heterogeneity (different cytoplasm colour). (**B**) In the blood the precursor expands and accumulates mutations, leading to different subclones (different colour of nucleus). (**C**,**D**) Lesions are seeded by malignant T-cells, where they progress malignancy and develop new subclones while disease further develops. (**E**) Some malignant cells may re-enter the circulation to self-seed into other lesions, increasing heterogeneity and disease progression. Reprinted with permission from ref. [27]. Copyright © 2020 American Society of Hematology.

In the majority of cases the malignant T-cell exhibits a CD4^+^ tissue resident effector memory phenotype [22]. The malignant T-cell might then disseminate via chronic antigenic stimulation leading to clonal proliferation [3]. It is believed that the first oncologic hit happens at the state of the mature tissue resident T-cell, but this understanding is recently challenged. It has been observed that malignant cells in lesions of the same patient can share the same T-cell receptor (TCR), and that a neoplastic clonotype can be found in the blood even months before its first skin occurrence [23]. With ongoing disease severity, there is a loss in TCR repertoire complexity and a shift towards a dominant clone [24]. Moreover, some of these clonotypes share the same TCR-gamma sequences but not TCR-beta and TCR-alpha, indicating an initial malignant transformation at the T-cell progenitor stage after TCR-gamma but before TCR-beta and –alpha reassembly [25,26]. Hence, a model of circulating and self-seeding T-cell clones is suggested (Figure 1): Early neoplastic clones may initially colonize the skin and other clones follow. Some early clones may mature and re-enter the circulation to self-seed into other lesions [23,25,26,27] leading to intra-patient heterogeneity.

High-throughput sequencing of the TCR-beta gene showed that MF patients with a tumour clone frequency (relative abundance of T-cell clones) in lesions less than 25% have a good prognosis for overall and progression-free survival, while this is inverse for tumour clone frequencies over 25%. However, such associations were not seen in patients with Sézary syndrome (SS), the leukemic variant of CTCL [8]. Interestingly, the most dominant TCR variable beta chain (TCR Vβ) family is found in frequencies comparable to SS patients [8,28]. Moreover, this specific TCR Vβ family is associated with *Staphylococcus aureus* (*S. aureus*) infections in a subset of CTCL patients [8,29]. This bacterium contributes to CTCL progression [30]. Because not all patients are colonized with *S. aureus* [14] and colonization with other commensals could protect against this pathogen [31,32], this could lead to a varying composition and prevalence of dominant malignant clones and therefore to inter-patient heterogeneity. Later, we will discuss the role of *S. aureus* in CTCL pathogenesis and its association with TCR Vβ in more detail.

## 3. Transcriptional Heterogeneity

A series of studies profiled the mutational landscape of CTCL [28,33,34,35,36]. However, an overall quite heterogeneous genomic picture is drawn, showing no well-defined alterations in fusion proteins, copy number, and somatic mutations. Gene modifications have been frequently found that are involved in specific cellular processes and signalling pathways [37]. Likewise, transcriptome analyses revealed a diverse picture: Litvinov et al. [9] used a targeted bulk RNA sequencing (RNA-seq) approach on mostly formaldehyde-fixed paraffin-embedded (FFPE) skin and a freshly snapped frozen specimen. The authors found differential heterogeneity between and even within the same patients that were sampled over the course of the disease with progressing MF stages. Unsupervised clustering showed intermixed groupings of CTCL stages and benign dermatoses. Similarly, cluster analyses failed to produce clear results to distinguish between early versus advanced disease stages as well as indolent versus aggressive CTCL samples.

On the single gene level, however, 75 transcripts were found to be upregulated across different CTCL stages, including inflammation-mediating genes like STAT and CD70. The authors underlined differential expression of *TOX*, *FYB*, *LEF1*, *CCR4*, *ITK*, *EED*, *POU2AF*, *IL-26*, *STAT5*, *BLK*, *GTSF1* and *PSORS1C2*, as these genes were already characterized in previous investigations using quantitative RT-PCR [38].

Focusing on advanced disease stages, T-cell related genes like *TOX* and *FYB* were upregulated. In early disease stages that are about to progress, *TOX*, *FYB* and other genes were overexpressed as compared to samples with an indolent course [8,9,11,39]. These genes are also associated with decreased disease-specific survival and might thereby serve as prognostic markers [11]. As CTCL is characterized by unregulated expanding T-cells, genes involved in TCR signalling are associated with a high tumour cell frequency [8]. *TOX* is not an exclusive CTCL marker, since it has been shown to be upregulated in primary cutaneous B-cell lymphomas and benign dermatoses as well [40,41]. However, Litvinov et al. pointed out that *TOX* expression in CTCL was several magnitudes higher than in benign dermatoses, climaxing in most advanced stages [9]. Targeted RNA-seq provides an effective method to investigate (low-abundant) transcripts that have been selected based on previous considerations [42,43], but naturally also provides only a restricted picture. Routinely generated FFPE specimen in clinics provide an extensive source of samples, especially in the case of diseases with rarer incidences. However, because RNA is usually degraded in FFPE samples, results might not reflect the true biological information [44]. A follow-up study addressing differences between FFPE and fresh samples and possible batch effects found no significant influences [45], although trends can be observed.

### 3.1. Single Cell RNA Sequencing Reveals Transcriptional Heterogeneity

With the advent of single cell RNA sequencing (scRNA-seq), more detailed insights into the behaviour of single cells and their states within a specific context have emerged. This can lead to improved onco-biological knowledge leading to better treatment and precision medicine. Therefore, scRNA-seq might be superior to bulk RNA-seq, although the latter still provides less noisy and more precise data [42,46,47,48]. In the case of MF, the tumour microenvironment is composed of a significant proportion of infiltrative reactive immune cells including CD8^+^ T-cells, regulatory T-cells (Tregs), dendritic cells (DCs), macrophages and mast cells. The infiltrative proportion decreases with disease progression and varies between patients [49,50]. Hence, bulk sequencing will determine average values of fluctuating percentages of malignant and benign cells and cannot assess unique transcriptome expression of tumour cells and its microenvironment [42,46]. Five studies utilized scRNA-seq for CTCL: Three investigated SS [51,52] with one study including a single MF subject [53] and two examined exclusively MF [12,54]. Gaydosik et al. [12] sequenced single cells of five advanced stage (IIB-IVA) MF patients. The lesions showed inter-patient heterogeneity as there was no overlap between cells from tumour samples or with cells from heathy controls revealed by clustering analysis. Focusing on lymphocytes, several gene expression clusters were found, mostly unique for specific MF samples. Enriched pathways in these clusters are associated with cell growth, proliferation and survival, translational reprogramming, metastasis, negative regulation of NK-mediated cytotoxicity against tumours, NK-cell signalling, and tumorigenic pathways known from other forms of cancers [12]. Noteworthy is the implication of a virus entry pathway which indicates exogenous triggers on CTCL.

**Figure 2:**
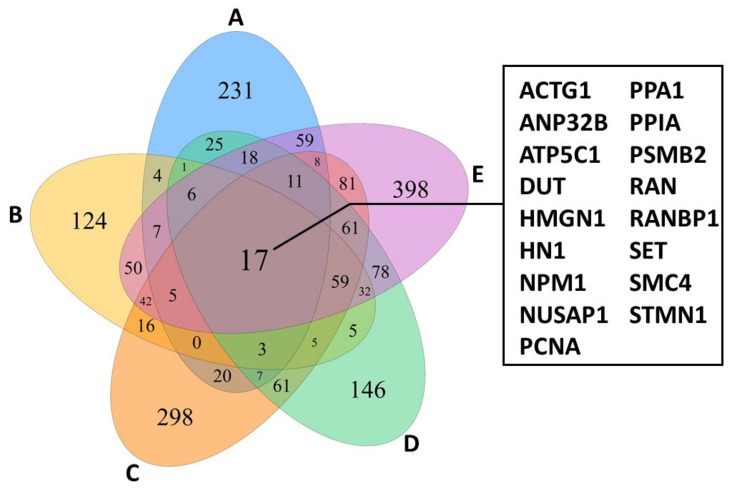
Venn diagram of proliferating lymphocytes from CTCL-lesions of 5 different patients (**A**–**E**) showing an overlap of 17 genes highly while only minor expression is observed in controls. The figure was created with specifications from [12].

Focusing only on actively proliferating lymphocytes of the five patients investigated revealed a shared panel of 17 genes that are overexpressed compared to healthy control individuals (Figure 2). Among other functions, these genes regulate cell cycle and survival, apoptosis, metabolism, and transportation of mRNA. Interestingly, the 17 genes were also expressed by *TOX*-positive cells and furthermore showed similar characteristics as the lymphocyte-tumour-clusters described above [12]. Hence, *TOX* might potentially serve as a diagnostic and prognostic marker [9,12]. Although the TCR was not sequenced, expansion of constant TCR-beta chain was observed in the MF gene expression clusters [12], indicating TCR clonality [8]. Concerning tumour-infiltrating lymphocytes (TILs) in the tumour microenvironment, all but two MF samples showed overlapping patterns, also with healthy controls. However, in MF samples, several checkpoint inhibitory receptors were heterogeneously expressed in CD4^+^ and CD8^+^ TILs [12]. Patient-dependent expression of multiple inhibitory receptors have been reported to hinder an efficient antitumor response and hampers the development of a treatment regimen, thus requiring personalized therapies [12].

Rindler et al. [54] showed intra-patient heterogeneity by investigating three body compartments of one advanced stage MF patient (skin, peripheral blood, and lymph node). Three clusters of malignant T-cells were identified by expression patterns and clonal TCR sequences. Two of the three malignant T-cell clusters exhibited comparable distributions of dominant TCR clonotypes between all investigated tissues, albeit one cluster was mainly present only in skin and blood. The third cluster showed clonality restricted to the skin, while the blood compartment was polyclonal. Differential gene expression revealed the malignant T-cell clusters as substantially heterogeneous. Pro-inflammatory markers not attributable to a specific T helper type were overexpressed in all tissues. However, in malignant skin cells mainly T helper 2 (Th2) cytokines were observed [54]. These cytokines have been linked to increased susceptibility for skin infections in CTCL [55] and might indicate microbial influence on skin cells.

To further investigate lineage relationships between malignant cells of the three body compartments, the authors used trajectory analysis. This computational method orders cells along branches based on similarities in their expression profile. As a result, lineage trees with branch points inferring cell fate are compiled. Mostly the resulting tree is 2-branched with a starting pre-branch (or root) [56,57]. Using this technique, the malignant skin and blood cells ordered at opposing branches. Skin cells accounted also for the starting pre-branch. Lymph node cells scattered alongside the whole trajectory. This indicates that lymph node cells share patterns with cells from both skin and blood. In the blood cell branch, loss of tissue retention can be observed while also retaining lymph node and skin homing ability [54]. Gene expression shows signatures for a central memory T-cell (Tcm)-like phenotype, which is typically attributed to SS [22]. Cells of the skin branch expressed markers associated with tissue retention and of tissue resident effector memory cells (Tem), which has been reported as the MF-typical tumour cell [22]. Pre-branched skin cells discriminated from post-branched skin cells by expression of Th2 cytokines as opposed to Th22, Th17 and Th1 cytokines as well as cell motility genes [54]. It was recently shown that tissue resident T-cells (Trm) could re-join the circulation upon reactivation and resemble closer Tcm than recently activated Trm. Mature Trm can differentiate into Tcm, Tem and again into Trm with preferred homing to tissue of origin [58]. In conclusion, the adaptive ability of MF tumour cells for long lasting skin residency and dissemination through the circulation might contribute to the heterogeneous nature of MF [54].

Likewise, investigation by Borcherding and co-workers on Sézary syndrome revealed substantial heterogeneity within malignant T-cells of a single patient [52]. Overexpressed pathways differed significantly between clusters of malignant cells. Besides the typical Tcm type usually seen in SS [22], one cluster expressed markers specific to regulatory T-cells (Tregs). Trajectory analysis suggested transition of *FOXP3* (Tregs) positive cells to either *GATA3* (T helper 2 cells) or *IKZF2* (Tregs) positive cells, which may depend on the tumour micromilieu [52]. T helper 2 phenotype is often accompanied with bacterial infections in CTCL [59]. Since peripheral blood was investigated and some malignant cells transitioned into T helper 2 like phenotypes, this could indicate that these cells came into bacterial contact at the skin and moved back into circulation.

Both studies by Rindler et al. [54] and Borcherding et al. [52] found interesting aspects of cell fates potentially driving the malignant CTCL cell to develop into different cell (sub-)types and thereby constituting to intra-patient disease heterogeneity. Since these two investigations are based on samples of only a single patient, the results need to be validated and extended in bigger patient cohorts.

Buus et al. [51] support the heterogeneous picture showing surface marker expression of naïve T-cells (Tn), stem-cell memory T-cells (Tscm), and expression patterns not fitting Tn, Tcm, and Tscm. Using targeted gene expression profiling of relevant T-cell genes, inter-patient patterns were observed. Cells of each patient grouped into clusters, suggesting disparate functional background of malignant cells. The authors reported a 5-genes panel expressed by most cells (*S100A4*, *S100A10*, *IL7R*, *CCR7*, and *CXCR4*). Three of which comprise known functions in growth and migration of malignant T-cells (*IL7R*, *CCR7*, and *CXCR4I*, while the other two are well known cancer related molecules that have yet not been associated with CTCL [51].

Herrera et al. [53] interrogated surface marker expression, TCR-repertoire and transcriptome of matched skin and blood from SS and one leukemic MF patient on the single cell level. Malignant T-cells were defined based on clonal TCR sequences and transcriptional and surface marker patterns distinct to non-malignant T-cells. Matched skin and blood samples shared dominant TCR sequences, suggesting involvement of both tissues in CTCL. Among differentially expressed genes were transcripts typically affected in CTCL like *CD158K/KIR3DL2* [60] and *TOX* [9,41]. Comparing matched malignant cells derived from the blood and the skin of the same patients revealed several distinct groups thereby displaying intra-patient heterogeneity. Blood-derived cells showed a higher level of clonal diversity than skin-derived cells. Trajectory analysis suggests that malignant cells from the blood transition into skin-derived cells. Together, these findings show a strong tissue dependency regarding transcriptional activity. In skin samples, several upregulated pathways were unmasked: T-cell activation, TCR ligation, mitogen-induced transcripts, and cell cycle. Vice versa, T-cell quiescence and markers for resting T-cells were upregulated in blood counterparts [53]. Strikingly, some of those genes were also outlined by Rindler et al. in blood of an advanced stage MF patient [54]. Moreover, skin-derived cells exhibited higher T-cell activation, reduced T-cell resting and more highly proliferating lymphocytes. As this was not seen in a healthy donor as well as in a psoriasis patient, stimulating activity from the CTCL skin microenvironment can be suspected [53]. Adding to this, several exogenous factors are discussed as CTCL initiating or promoting [16]. Since phylogenetic analysis based on inferred genetic abnormalities showed no clear linear relationship between skin and blood sub-clones, the skin microenvironment seems to serve as driving factor in stimulation of malignant cells and the clonal expansion of few and specific sub-clones [53]. Because varying proportions of colonizing microbiota on CTCL patients were reported [14], this could lead to inter-patient heterogeneity additive to intra-patient heterogeneity demonstrated by Herrra et al. [53]. On the other hand, decreased diversity of clonal diversity in skin cells in contrast to matched blood counterparts might indicate a homogenization of transcriptional activity by skewing of malignant cells from the blood to the skin compartment. In conclusion, microbial colonization might lead to skewing of malignant cells in the skin which can differ between CTCL lesions and between CTCL patients depending on the specific microbiome present.

### 3.2. Single Cell Transcriptome Signatures Suggest Microbial Influence on CTCL Heterogeneity

Taken together, several single cell transcriptomic signatures suggest exogeneous/microbial influence on CTCL heterogeneity. Gaydosik et al. found an upregulated pathway of endocytic virus entry. Dysregulation of epithelial-mesenchymal transition as well as skin-barrier dysfunction may reflect increased susceptibility to infections [12]. Additionally, typical cytokines for the T helper 2 (Th2) phenotype were upregulated in malignant skin cells in contrast to matched malignant cells from the blood and lymph node of the same patient [54]. Th2 cytokines were found to be associated with impaired production of S100 proteins [55]. S100 proteins contribute to tumorigenic processes in diverse cancers and expression patterns can be stage- and cancer subtype-specific. S100 inhibitors already are in clinical trials for e.g., melanoma [61]. In addition, some S100 family members are so called antimicrobial peptides (AMPs) with bactericidal activity [62]. In the scRNA-seq studies review, several S100 family members were differentially expressed [12,51,52,54]. Among them, S100A4, S100A8, S100A9 and S100A12 have known bactericidal activity or are implicated with bacterial infections [63,64,65,66]. Indeed, CTCL exhibits an impaired skin barrier and deficient expression of several AMPs leads to increased susceptibility for skin infections and bacterial toxins may play a major role in driving disease progression [55,59,67,68]. Since microbial colonization is not consistent between CTCL patients, as shown in an early study by Axelrod et al. [14] and extended by two investigations utilizing microbiomics [20,21], differential microbial colonization may account for transcriptional heterogeneity.

## 4. Microbiota and CTCL

### 4.1. Skin Barrier Dysfunction

Different exogenous triggers like skin resident microbiota are being discussed as CTCL provoking and/or promoting factors [16]. In a CTCL mouse model developed by Fanok et al. [34], disease progression was depended on microbial triggers. Mice housed under germ-free conditions were significantly less CTCL symptomatic [34]. Hence, there seems to be crosstalk between CTCL cells and/or the tumour microenvironment and microbiota. Indeed, MF presents with a skin barrier dysfunction [59]. It has been shown that malignant T-cells secrete galectins in CTCL [69]. This is a class of proteins with functions in several biological activities like cell proliferation and implications in inflammatory skin diseases and cancers [70,71,72]. In CTCL, galectins might induce morphological and histopathological changes via epidermal hyperproliferation, disorganized keratinocyte stratification and decreased attachment between the epithelial and mesenchymal layer [69]. Moreover, during CTCL progression, a shift in the inflammatory tumour micromilieu can be seen. In early stages, CTCL lesions typically present with a high abundance of benign reactive T helper 1 (Th1) cells, thereby expressing according Th1-markers. As the disease progresses, a decline in Th1 cells and its markers are observed. Concomitant, malignant T-cells and T helper 2 (Th2) cells increase, leading to a Th2 dominated inflammatory milieu [73]. Th2 cytokines like interleukin (IL)-4 and IL-13 suppress the appropriate expression of skin produced antimicrobials peptide (AMP) [74]. This effect is even more pronounced than in atopic dermatitis (AD) and psoriasis, which are other inflammatory skin diseases that often are overgrown by bacterial pathogens [55,59,67]. As already stated above, AMPs are differentially expressed in CTCL on the single cell level as well [12,51,52,54].

Further indication for the influencing effect of AMPs on CTCL progression is added by the discovery of geographic patterns of CTCL cases. They indicate a link between sunlight exposure leading to vitamin D expression which in turn upregulates AMPs: Demographic data from CTCL patients in Texas, USA, shows that several communities had five to twenty times higher incidences than the expected rate for the population [75]. On the other hand, only few communities were completely spared by CTCL. Among them are areas near El Paso, which is one of the sunniest cities in the USA with an annual sunshine of 84%. Sun exposure might therefore be a protective or a therapeutic factor [75,76]. Noteworthy, sun exposure has been shown to reduce risk for other non-Hodgkin lymphomas as well [77]. As it is well known, sunlight exposure leads to vitamin D production [78]. Vitamin D is already in use to treat other inflammatory skin diseases like AD [79] and psoriasis [80]. Moreover, vitamin D emerged as a possible cancer preventive agent which raises the question of its role in CTCL [81]. CTCL patients have vitamin D deficiency with a comparable prevalence to other cancer patients [82]. Under non-inflammatory conditions, vitamin D induces the expression of the cathelicidin LL-37, an AMP with strong antibacterial, anti-biofilm, antifungal and antiviral actions. In return, microbial proteases (e.g., released by *Staphylococcus aureus*) might cleave LL-37 into inactive fragments [83].

In summary, CTCL presents with a dysfunctional skin barrier and reduced AMP production (either due to cytokine shifts during disease progression and/or environmental factors). This results in an enhanced skin permeability leading to greater susceptibility for skin infections [16,69].

### 4.2. Microbiome on CTCL Lesions

Many common skin diseases are associated with changes in the microbiota, which is termed dysbiosis [19]. While the overrepresentation of *Staphylococcus aureus* on CTCL lesions has been reported [84] and will be discussed later, other microorganisms may also play a role in disease progression. For example, interactions between skin commensals can comprise of competitively excluding one another, or synergies for mutual benefits. Especially interactions with *S. aureus* have been studied [19]. An early study assessed the relative abundance of microbes on CTCL involved skin using traditional culture-based methods [14]. However, more than 99% of all microorganisms still cannot be isolated by bacterial cultures even today [85]. The vast majority of microbial isolates belong to only four phyla and hence uncultured microbes are referred to as “microbial dark matter”. Culture-based approaches select for appropriate microorganisms thereby underestimating the total diversity of the community [86]. There are several culture-independent methods to study the microbiome, each with their own advantages and disadvantages [87,88]. The most used method is amplicon sequencing, where marker genes like the 16S ribosome DNA (rDNA) with conserved regions are amplified. The process of 16S amplicon sequencing requires only low biomass and is not influenced by host DNA contamination but comes with PCR bias and a limited resolution. Whole-metagenomic shotgun sequencing (WMS) on the other hand is affected by host DNA but provides deeper resolution down to the microbial strain level and holds potential for functional analysis, e.g., screening for enriched pathways or virulence factors and antibiotic resistance genes [88,89].

**Figure 3:**
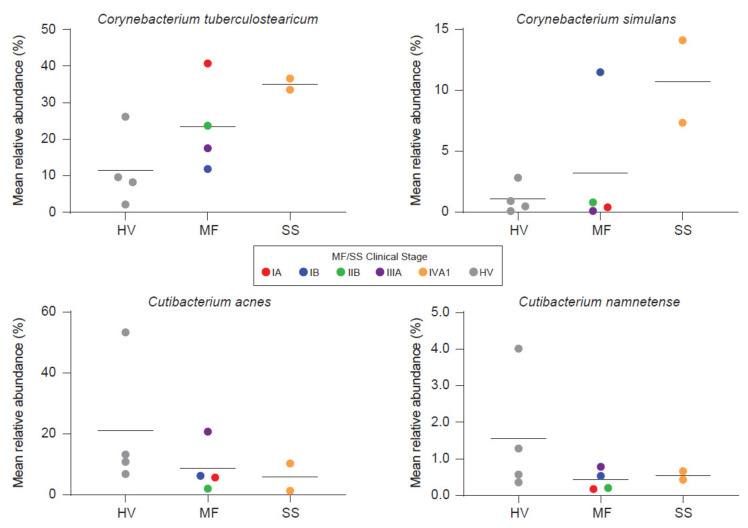
Besides *Staphylococcus aureus*, other organisms may also be linked with CTCL. Harkins et al. [20] found two corynebacteria to trend higher, whereas two cutibactactial species exhibited decreased abundances on CTCL lesions. Bacterial shifts seem to correlate with disease stage. HV = Healthy Volunteer. Reprinted with permission from ref. [20]. Copyright © 2021 Elsevier.

Salava et al. used 16S and WMS to investigate the microbiome on early stage CTCL lesions while using non-lesional skin of the same patients as an internal control [21]. WMS data delivered higher resolution in the genus of propionibacteria as compared to 16s sequencing and subsequent analysis is therefore based on WMS. The authors observed patient and body site specific variation, which is to be expected [90]. No differences were found in terms of community diversity [21]. However, the presented data suggests a trend towards stable communities in non-lesional skin and an unpreserved microbiome composition on CTCL lesions. Hence, a dysbiotic flora might exist on lesional skin. Ten bacterial species were identified to be more abundant on non-lesional skin. Only two had been associated with cutaneous diseases before. These are *Serratia spp.* and *Pseudomonas spp.*, that affect the skin in nosocomial infections and immunocompromised patients [21]. Others reported overrepresentation of *Staphylococci* on CTCL lesions using 16S sequencing. Moreover, the phylogenetic diversity was decreased, indicating a distinct microbiome as compared to healthy volunteers and psoriasis patients [30]. Psoriasis as an inflammatory disease is, among others, frequently confounded with early-stage MF [5]. Compared to atopic dermatitis (AD), decreased community diversity correlated significantly with flare and recovered post flare. Staphylococci increase during AD flare, whereas relative abundances of other microbial genera vary across AD disease states, pointing to the complex relationship present in microbial communities [91,92]. On the other hand, Harkins et al. [20] found no differences in the microbial diversity investigating early and advanced stage MF and SS patients compared to age-, sex- and sampling site-matched healthy volunteers. Fungal and viral abundances were low and showed no differences to healthy volunteers, contradicting hints about viral implications in CTCL [93]. Interestingly, *S. aureus* also showed no differences whereas other commensal staphylococcal species trended higher in MF but not in SS. Nonetheless, principal coordinate analysis showed separation of samples from healthy volunteers and advanced stage CTCL. This may be driven by overrepresentation of two corynebacterial species and decreased abundances of two cutibacterial species. Bacterial shifts, furthermore, seem to correlate with disease stage (Figure 3).

However, statistical significance was not reached, likely due to the small sample number [20]. *C. tuberculosearicum* can upregulate and/or induce inflammatory responses in keratinocyte derived cell lines in vitro and may contribute to cutaneous malignancies [94]. Besides this pathogenic role, corynebacteria also possess the ability to shift *S. aureus* towards commensalism via quorum sensing [95]. Furthermore, staphylococci like *S. epidermidis* and *S. hominis* are capable of controlling *S. aureus* virulence either indirectly through quorum sensing or directly via antimicrobial action in AD [31,32,96]. Under healthy conditions, *S. epidermidis* provides antagonistic action against *S. aureus* leading to negatively correlated colonization rates between these two bacteria [97]. Under disease/non-homeostatic skin flora conditions like AD, *S. aureus* abundance can increase substantially [92]. In parallel, there might also be an increase in *S. epidermidis*, possibly reflecting an attempt to control the vast overrepresentation of *S. aureus* [92].

### 4.3. Staphylococcus aureus

Investigations show that CTCL lesions are often colonized by S. aureus [84]. This bacterium possesses a large repertoire of virulence factors [98]. Staphylococcal alpha-toxin (a-haemolysin) induces cell death in CTCL-derived benign cells leaving their malignant counterparts unharmed. Malignant cells dispose of several resistance mechanisms to a-haemolysin, favouring the survival of CTCL tumour cells upon toxin presentation [99]. Additionally, this toxin inhibits T-cell mediated cytotoxic anti-cancer responses, leading to tumour escape and continued persistence of respective cells [100]. Besides haemolysins, *S. aureus* produces staphylococcal enterotoxins (SEs) that can act as so-called superantigens [101]. Superantigens do not need to be processed by antigen presenting cells (APCs) but rather bind directly to the variable beta chain of the TCR and to the major histocompatibility complex class II (MHC II) outside of the antigen binding groove, thereby triggering clonal expansion and an upregulation of pro-inflammatory cytokines [102]. In a study investigating TCR clones in a big CTCL cohort via high-throughput sequencing, the most abundant TCR Vβ family was TRBV20 [8]. The staphylococcal toxin toxic shock syndrome toxin-1 (TSST-1) binds specifically to TRBV20 [103]. Moreover, TRBV20 expansion has been shown to correlate with TSST-1 level in clinical CTCL isolates. Besides TSST-1, other SEs were detected as well but not linked to TRBV20 ratios [29]. However, in another CTCL cohort, only staphylococcal enterotoxin A (SEA) but not TSST-1 or other SEs were found to be present on patient skin. SEA and staphylococcal enterotoxin E (SEE) were the only staphylococcal toxins to elicit disease-stimulating activity in SS patient-derived tumour cells in vitro [17]. Hence, this conflicting data might be caused by (i) other SEs than TSST-1 associating with TRBV20, (ii) specific host-pathogen interactions [104] that differed between the CTCL cohorts and/or (iii) microbe-microbe interactions leading to an altered expression of virulence factors [19]. Taken together, clonal expansion of malignant cells [50], loss of TCR repertoire complexity and TCR Vβ skewing [24] during CTCL disease course might (i) reflect the self-seeding mechanism described previously [27] and (ii) may be enhanced and/or triggered by superantigenic activity of colonizing microbes [68] that can also act specifically among themselves [19] and with the hosts skin immunity [104].

**Figure 4:**
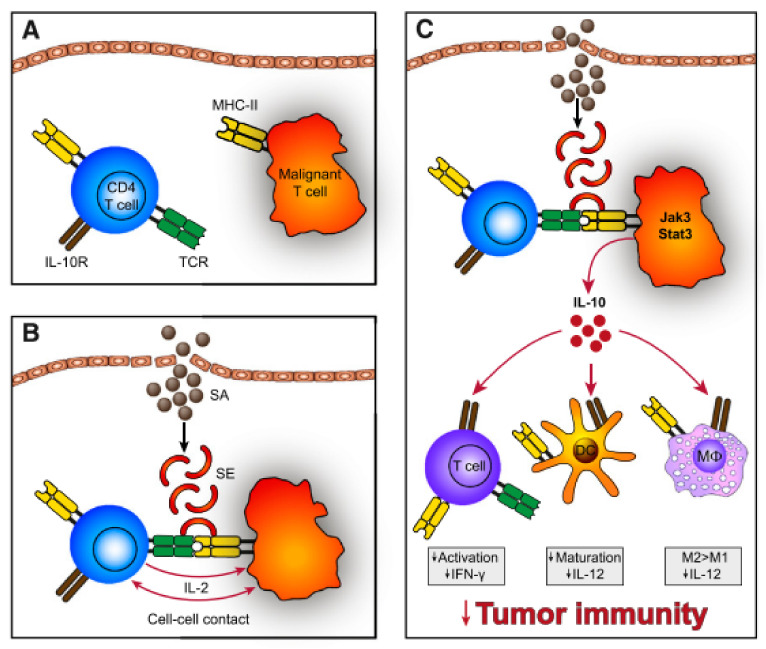
(**A**) Malignant T-cells often express a monoclonal TCR-V-beta chain with decreased function of the whole TCR complex. Hence, SEs do not stimulate the TCR directly but through activation of benign T-cells. (**B**) SEs bind to MHC II expressed on malignant T-cells, which crosslink to TCR of benign T-cells. Subsequently, establishing T-T-cell interaction and IL-2 is expressed. (**C**) These signals induce IL-10 expression, which dampens the immune response via impaired maturation of DCs, repressed expression of Th1 cytokines (interferon-g, IL-12), inhibition of T-cell activation and promoted development of M2 macrophages. Reprinted with permission from ref. [105]. Copyright © 2014 American Society of Hematology.

In Vitro, SEs can stimulate CTCL disease activity via cell-cell contact of malignant and benign T-cells [105], which may act in a T-T-cell interaction manner by T-cells bearing MHC II [106,107] (Figure 4). Cross-linked TCR-MHC II prompts the benign T-cells to produce IL-2 which in turn upregulates the Janus kinase 3/signal transducer and activator of transcription 3 (Jak3/Stat3) pathway. Activation of Jak3/Stat3 in malignant cells leads to secretion of soluble factors, supressing cell mediated cytotoxicity in benign immune cells and promoting aggregation of immunosuppressive regulatory T-cells. Furthermore, aberrant Jak3/Sat3 induces overexpression of IL-10, which dampens immunity by several means: Downregulation of Th1 responses (interferon-g, IL-12), favouring anergic and immunosuppressive T-cells, repression of DC maturation and promotion of immunoregulatory M2 macrophages [105]. In addition, SEs also activates the Stat5 protein and upregulates factors contributing to shifting the Th1 milieu to a Th2 dominated milieu [108]. The latter is usually seen in advanced stage CTCL [73]. Moreover, staphylococcal toxins disturb elimination of malignant cells by cytotoxic T-cells and induce upregulation of the regulatory T-cell marker FOXP3 [100,109]. Regulatory T-cells suppress autoimmunity leading to self-tolerance [110]. Together this might result in a reduced capacity of the immune system to clear malignant cells from tissues.

In conclusion, these results hold compelling evidence for the role of *S. aureus* on the progression of CTCL.

Because *S. aureus* is also a member of the physiological skin flora [19], specific strains might be responsible for CTCL progression. Indeed, SEs are a group of several toxins [101] and different *S. aureus* strains dispose of different SEs [98]. While some SEs showed CTCL promoting activities (i.e., SEA and SEE), not all SEs act as CTCL stimulatory agents (e.g., TSST-1) [17]. Strain-level specificity was already shown to be related to AD and psoriasis at their disease severity [91,111]. Hence, not only the presence of *S. aureus*, but also the specific strain may be responsible for disease stimulating actions. Since TSST-1 was associated with the specific TCR Vβ family TRBV20 [8,29], but did not elicit stimulating activites in vitro [17], these conflicting results warrant further investigations.

### 4.4. S. aureus Eradication

Because hospitalized CTCL patients suffer from recurrent staphylococcal sepsis, systemic antibiotic treatment is often applied. However, as a side benefit, rapid clinical improvement of CTCL burden in some patients has been observed [29,68]. In an early study, absence of *S. aureus* after antibiotic treatment over several months was linked to a decline in disease severity in 2 patients [112]. Another study reported skin improvement in the majority of 33 CTCL patients who are *S. aureus* positive after administering oral and topical (nares) antibiotic agents over a course of several months. Response to treatment was observed over all disease stages [84].

Lindahl et al. [113] showed that decreased CTCL severity and *S. aureus* colonization after antibiotic treatment is linked to a decrease in malignant T-cells and normalization of several markers and pathways typically upregulated in CTCL: Systemic antibiotics were applied to six advanced stage MF patients and two SS patients over 24 days, who did not respond to standard treatment. Eradication of *S. aureus* resulted in significant disease improvement and a decrease in malignant T-cells depicted as the relative frequency of the most dominant TCR-beta clone. Microarray gene expression analysis revealed normalization of markers for cell proliferation, inflammatory response, neoplasia and the Stat3 and IL-2 pathways compared to healthy controls, even one month after cessation of antibiotic treatment. In isolated primary malignant cells, Staphylococcal enterotoxin A (SEA) led to an activation of Stat3 and IL2-Receptor, while antibiotics showed no effect. Together these findings strongly suggest that *S. aureus* directly or indirectly stimulate CTCL tumour cells, and that antibiotic mediated disease relief is depended [113]. Unfortunately, *S. aureus* re-emerges quickly in most patients after termination of systemic antibiotic application. This finding emphasizes the clinical need for appropriate management of CTCL infections. Lifelong antibiotic therapy is not feasible due to antibiotic side-effects and the risk of new antibiotic resistances [114]. One third of CTCL patients colonized by *S. aureus* are methicillin-resistant S. aureus (MRSA) [115], hence non-antibiotic treatment options are warranted. Duvic et al. [116] reported a new treatment regimen combining systemic antibiotics, antiseptic whirlpool bathing, corticosteroids with alternating topical antibiotics as well as antiseptic creams applied to ulcers, resulting in profound clinical response [116].

## 5. Conclusions

CTCL presents with substantial clinical and transcriptional heterogeneity that might originate from adaptive and functional plasticity of malignant T-cells [7,10,53,54]. However, *S. aureus* can highly influence disease course and eradication of this pathogen may result in profound clinical improvements [68,113]. CTCL patients have a dysfunctional skin barrier and are susceptible to infections [67]. On the single cell level, several pathways and differential expression of AMPs strongly suggest exogenous impact of pathogens [12,51,52,54]. In particular, a consistently higher activation score of skin T-cells in comparison with their blood counterparts and benign skin T-cells indicate the cutaneous microenvironment to promote expansion of malignant T-cells [53]. Some data suggest that microbial stimuli could lead to a homogenization of the transcriptional profile of malignant cells [53]. *S. aureus* seems to foster expansion of a specific TCR Vβ family [8], thereby skewing malignant cells to a few clones. Since *S. aureus* constitutes only a minor part of the complex microbiome on human skin with many other microbes orchestrating human health and disease [19], other microbes may also be implicated in CTCL. Only few microbiome studies dealing with CTCL have been carried out so far. They deliver not yet a consistent picture but rather seemingly display microbe colonization to be patient and CTCL stage dependent [20,21]. Since some microorganisms have protective properties for the host [31,32,96], the specific microbial community composition on CTCL lesions can be crucial for the virulence of pathogens. Taken together, while specific microorganism like *S. aureus* could lead to a homogenization of transcriptional response, observed transcriptional heterogeneity might at least in part also be attributed to differential microbial colonization between CTCL lesions and between CTCL patients. Of course, it could also be speculated that transcriptional heterogeneity of CTCL itself leads to the observed microbiome patterns. Thus, there could also be a reciprocal influence of the microbiome and transcriptome in CTCL. Studies investigating mechanistic relationships are needed to clarify this “chicken-and-egg” situation.

There is no curative therapy available until today and optimal treatment remains to be elucidated [117]. Early stages have comparably good 5-year overall survival and thus maintenance of stable disease is important [118]. Additionally, CTCL patients frequently suffer from pain, pruritus, as well as disfigurement consequently affecting health-related quality of life [119]. Shaping the microbiome as personalized skin precision medicine might be an option to relieve symptoms and prevent disease progression [120]. Recently, bacteriotherapy to control *S. aureus* in AD patients was successfully tested in a phase 1 clinical trial presumably leading to improvement in eczema severity [121]. However, understanding the complex microbiome patterns of CTCL is still in its infancy. Strain level diversity of colonizing pathogenic and protective microorganisms on MF lesions remains to be illuminated to add knowledge about possible treatment regimen. In addition, investigating temporal microbial community shifts might be of interest as suggested by associations with AD opposed to healthy skin [92,122,123,124]. Owing to first hints about distinctive characteristics between cutaneous malignancies as well as between CTCL stages [20,30], microbial approaches could also be utilized as prognostic and diagnostic tools [120,125]. Further studies are needed to shed light on the relationship between CTCL and its microbiome. The high level of transcriptional heterogeneity on the single cell level emphasizes the need to expand the given results in bigger cohorts. Research is warranted to understand the (reciprocal) influence of differential microbial colonization on single tumour cells and its microenvironment.
